# Caffeine Mediates Sustained Inactivation of Breast Cancer-Associated Myofibroblasts via Up-Regulation of Tumor Suppressor Genes

**DOI:** 10.1371/journal.pone.0090907

**Published:** 2014-03-03

**Authors:** Mysoon M. Al-Ansari, Abdelilah Aboussekhra

**Affiliations:** 1 Department of Microbiology, Faculty of Science and Medical Studies, King Saud University, Riyadh, Saudi Arabia; 2 Department of Molecular Oncology, King Faisal Specialist Hospital and Research Center, Riyadh, Saudi Arabia; University of Hawaii Cancer Center, United States of America

## Abstract

**Background:**

Active cancer-associated fibroblasts (CAFs) or myofibroblasts play important roles not only in the development and progression of breast carcinomas, but also in their prognosis and treatment. Therefore, targeting these cells through suppressing their supportive procarcinogenic paracrine effects is mandatory for improving the current therapies that are mainly targeting tumor cells. To this end, we investigated the effect of the natural and pharmacologically safe molecule, caffeine, on CAF cells and their various procarcinogenic effects.

**Methodology/Principal Findings:**

We have shown here that caffeine up-regulates the tumor suppressor proteins p16, p21, p53 and Cav-1, and reduces the expression/secretion of various cytokines (IL-6, TGF-β, SDF-1 and MMP-2), and down-regulates α-SMA. Furthermore, caffeine suppressed the migratory/invasiveness abilities of CAF cells through PTEN-dependent Akt/Erk1/2 inactivation. Moreover, caffeine reduced the paracrine pro-invasion/−migration effects of CAF cells on breast cancer cells. These results indicate that caffeine can inactivate breast stromal myofibroblasts. This has been confirmed by showing that caffeine also suppresses the paracrine pro-angiogenic effect of CAF cells through down-regulating HIF-1αand its downstream effector VEGF-A. Interestingly, these effects were sustained in absence of caffeine.

**Conclusion/Significance:**

The present findings provide a proof of principle that breast cancer myofibroblasts can be inactivated, and thereby caffeine may provide a safe and effective prevention against breast tumor growth/recurrence through inhibition of the procarcinogenic effects of active stromal fibroblasts.

## Introduction

Breast cancer remains the leading cause of morbidity and second-leading cause of death in women worldwide [1]. Breast carcinogenesis is a complex process involving molecular and functional alterations in both the epithelial compartment and its microenvironment. Several lines of evidence indicate that cancer-associated fibroblasts (CAFs), which constitute a major portion of the reactive tumor stroma, actively participate in tumor growth, invasion and metastasis [2]–[4]. Indeed, a large amount of data has emerged showing cancer-promoting function of these cells through paracrine effects that escort tumor cells through all the carcinogenesis steps. This involves many signaling proteins that transmit the message in both directions, allowing cooperative crosstalk between cancer cells and their stroma. It has been clearly shown that stromal fibroblasts present in invasive human breast carcinomas promote tumor growth and angiogenesis through elevated SDF-1 and vascular endothelial growth factor A (VEGF-A) secretion [5], [6]. Several other chemokines, growth factors and matrix metalloproteinases (MMPs) also participate in this stromal fibroblast-related activation of tumor growth and spread [3], [7].

Thereby, it became clear that an efficient cancer therapy should take into account the presence of stromal cells, which could be responsible for resistance to treatment and also the recurrence of tumor cells [8]. To respond to such vital problem some bioactive dietary components could be of great therapeutic value. Indeed, several natural products are already in use as therapeutic drugs or are currently involved in late-phase clinical trials [9].

Caffeine (1,3,7-trimethylxanthine), a natural purine alkaloid found in coffee, tea, and cacao, is the most widely consumed psychoactive substance in the world. Caffeine has different pharmacological actions. Recently, many studies reported that caffeine has anti-cancer properties through the induction of apoptosis and suppression of cell proliferation in several cancer types [10]–[15].

Therefore, we questioned here whether caffeine has the potential to suppress the procarcinogenic effects of active breast cancer-associated fibroblasts. The present data provide clear evidence that caffeine can permanently inactivate breast stromal myofibroblasts, and consequently inhibits their paracrine procanrcinogenic effects.

## Materials and Methods

### Cells, Cell Culture and Chemicals

Breast fibroblast cells were obtained and cultured as previously described [16]. Breast tissues were obtained from patients who underwent surgery at the King Faisal Specialist Hospital. Signed informed consent was obtained from all the patients under the Research Ethical Committee Project number RAC#2031091. HUVEC and MDA-MB-231 cells were obtained from ATCC and were cultured following the instructions of the company. All supplements were obtained from Sigma (Saint Louis, MO, USA) except for antibiotics and antimycotics solutions, which were purchased from Gibco (Grand Island, NY, USA). Caffeine (Sigma) was dissolved in water.

### Cellular Lysate Preparation and Immunoblotting

This has been performed as previously described [17]. Antibodies directed against alpha smooth muscle actin (α-SMA), transforming growth factor beta 1 (TGF-β1, 2AR2, vascular endothelial growth factor A (VEGF-A), Stromal**-**derived factor**-**1 (SDF-1) and interleukin-6 (IL-6) were purchased from Abcam (Cambridge, MA); matrix metalloproteinase 2 (MMP-2), hypoxia inducible factor-1α (HIF-1α), Akt and phospho-Akt (193H12), Erk1/2 and phospho-Erk1/2 (THR202/TYR204) from Cell Signaling (Danvers, MA), p16^INK4a^ and Caveolin-1 from BD Biosciences (San Jose, CA); p21 (F-5), p53 (DO-1), PTEN (A2B1) and glyceraldehydes-3-phosphate dehydrogenase (GAPDH, FL-335) were purchased from Santa Cruz (Santa Cruz, CA).

### siRNA Transfection

CAF-180 cells were transfected with PTEN-siRNA and non-targeting siRNA (Thermo-Scientific) using RNAiFect transfection reagent, as recommended by the manufacturer.

### RNA Purification and qRT-PCR

This has been performed as previously described [18]. The respective primers were:

***β-actin***: 5′-CCCAGCACAATGAAGATCAAGATCAT-3′ and 5′ ATCTGCTGGAAGGTGGACAGCGA-3′;

***VEGF-A***: 5′-CCCACTGAGGAGTCCAACAT-3′ and 5′- TTTCTTGCGCTTTCGTTTTT-3′.

***SDF1***∶5′-GATTGTAGCCCGGCTGAAGA-3′; and 5′-TTCGGTCAATGCACACTTGT-3′.

***MMP2***∶5′-CATGTCGCCCCTAAAACAGA-3′ and 5′-CCATCAAACGGGTATCCATC-3′.

***IL-6***∶5′-ATGAACTCCTTCTCCACAAG-3′ and 5′-ACATTTGCCGAAGAGCCCTCAG-3′

### ELISA Assay

Conditioned media from cell cultures, either treated with caffeine or not, were harvested and ELISA was performed according to the manufacturer’s instructions (R&D Systems). The OD was used at 450-nm on a standard ELISA plate-reader.

### Quantification of Protein and RNA Expression Levels

The expression levels of the immunoblotted proteins were measured using the densitometer (BIO-RAD GS-800 Calibrated Densitometer) as previously described [17].

### Chemotaxis and Invasion Assay

Cell migration and invasion was evaluated using the 24-well BD BioCoat Matrigel Invasion Chamber as per the manufacturer guideline (BD Bioscience). In brief, 0.75 mL of migration buffer (serum-free media) or chemo-attractants (serum-containing media) were added to the lower chambers. Cells were washed 3 times in migration media and 2–4×10^5^ cells were added to the upper wells separated by an 8 micron pore size PET membrane with a thin layer of matrigel basement membrane matrix (for invasion) or without (for migration). The membranes were stained with a Diff Quick stain (Fisher Scientific) after removing the non-migrated cells from the top of the membrane with Q-tips. After air drying, the membranes were cut and mounted on slides with oil and cells that had migrated to the underside of the filter were counted using light microscope (Zeiss Axio Observer) in five randomly selected fields (magnification; 40x). Each assay was performed in triplicate. The results were expressed as mean SD of migrating cells per fields counted.

### HUVEC Endothelial Tube Formation Assay

The endothelial tube formation assay was performed using the Chemicon *in vitro* Angiogenesis Assay Kit. Wells in a 96 well plate were coated with ice cold EC Matrix Gel Solution in the EC Matrix Diluent Buffer. After solidification of the matrix at 37°C, HUVEC cells were seeded onto the polymerized EC Matrix at a concentration of 5×10^4^ cells in 50 µL of RPMI media per well. Conditioned media, complete media (as positive control) or serum-free media (as negative control) were immediately added after plating HUVEC cells for a final 1∶1 ratio of RPMI media to conditioned media. The number of tubule branches and cavities were photographed and counted after 18 hr of incubation.

### Assessment of SA- β -gal Activity

Endogenous senescence associated β-galactosidase activity was assessed histochemically. Cells were fixed in 0.5% glutaraldehyde in PBS for 15 min, and then permeabilized with 0.02% Nonidet P-40 (NP-40) with 0.1% sodium deoxycholate for 15 min, followed by incubation (overnight at 37°C) in a 1 mg/ml solution of X-Gal substrate (5-bromo-4-chloro-3-indolyl-b-D-galactopyranoside) with 5 mM potassium ferricyanide and 2 mM magnesium chloride together with the above-mentioned detergents, at an acidic pH (6.0). The proportion of β-galactosidase (β-gal) positive cells was assessed in a total count of 500 cells.

### Conditioned Media

Cells were cultured in media +/− serum, and then the media were collected and centrifuged. The resulting supernatants were either used immediately or frozen at −80°C until needed.

### Statistical Analysis

Statistical analysis was performed by student’s t-test and *p*<0.05 was considered as statistically significant.

## Results

### Caffeine Up-regulates the Tumor Suppressor Proteins p16, p21, p53 and Caveolin-1 in Active Breast Stromal Fibroblasts

**Figure 1:**
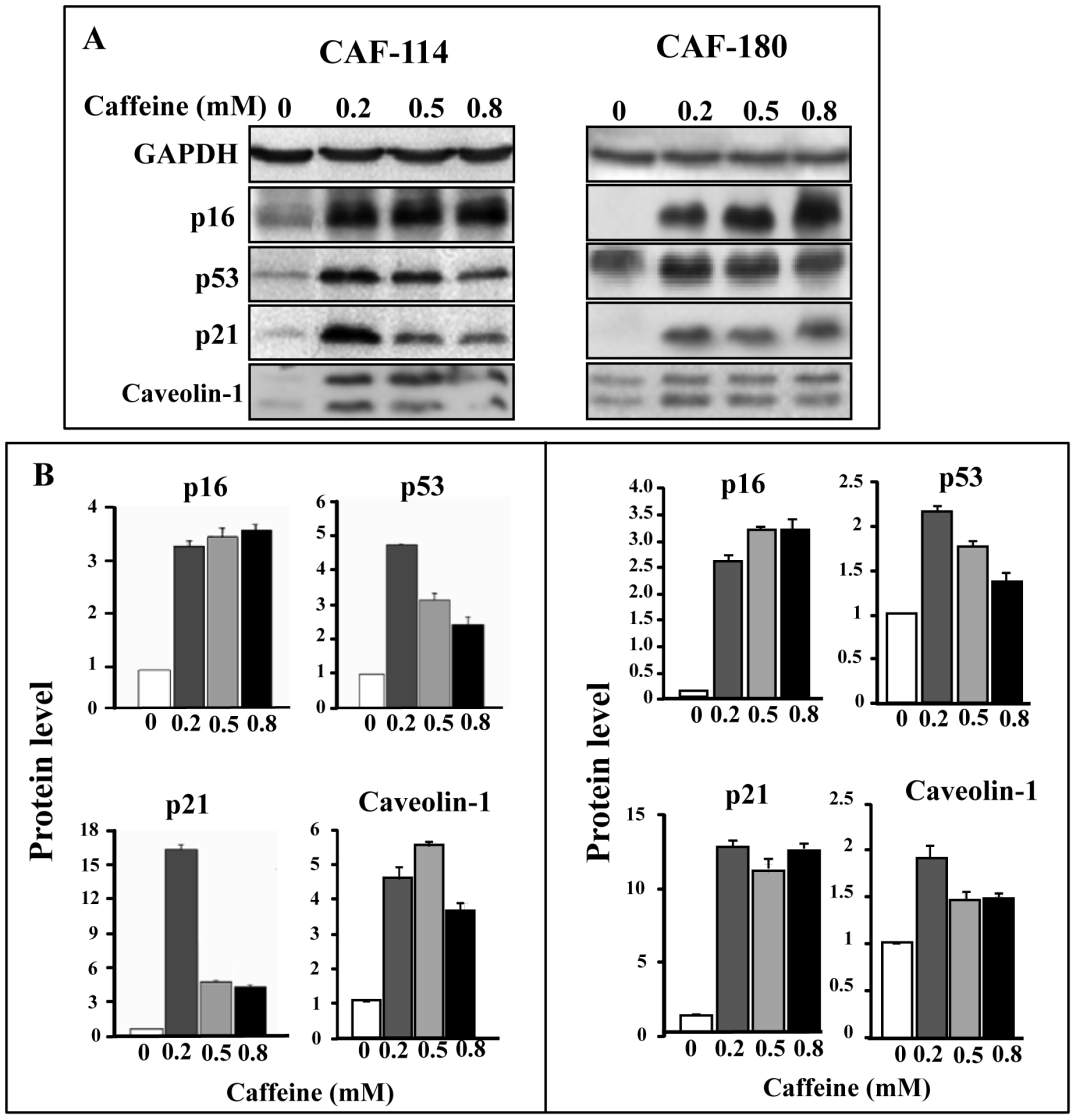
Caffeine up-regulates the expression of p16, p21, p53 and Cav-1. Cells were either sham treated or challenged with the indicated caffeine concentrations for 1 µg of proteins were used for immunoblotting analysis using the indicated antibodies. (A) Immunoblots. (B) Histograms show the expression levels of the indicated proteins. The values were determined by densitometry and normalized against GAPDH. Error bars represent means ± S.D.

It has been previously shown that the tumor suppressor proteins p16^INK4A^ (p16), p53, p21^WAF1^ (p21) and Cav-1 are down-regulated in CAFs and enhance the pro-carcinogenic paracrine effects of these cells [2], [18]. Therefore, the major aim of the present study was to explore the possible up-regulation of these genes in breast stromal myofibroblasts using caffeine. To this end, the primary breast cancer-associated fibroblasts CAF-114 and CAF-180 were treated with different doses of caffeine (0, 0.2, 0.5 and 0.8 mM) for 1 hr, and then were harvested and whole cell lysates were prepared for immunoblotting analysis using specific antibodies. Figure 1A shows clear up-regulation of p16, p21, p53 and Cav-1 in response to all caffeine doses in both cell cultures. Figure 1B shows that the maximum fold of induction for most of these genes was reached in response to 0.2 mM in both CAF-114 and CAF-180. In response to higher doses (0.5 mM and 0.8 mM), p16 level further increased in both cell cultures but only slightly, while the levels of p53 and Cav-1 were slightly reduced (Figure 1B). For p21 level, no significant change occurred in CAF-180, while a strong decrease was observed in CAF-114 in response to 0.5 and 0.8 mM of caffeine (Figure 1B). This indicates that 1 hr of caffeine treatment at low dose (0.2 mM) up-regulates the tumor suppressor proteins p16, p21, p53 and Cav-1 in breast CAF cells.

### Caffeine Represses Active Stromal Fibroblasts

**Figure 2:**
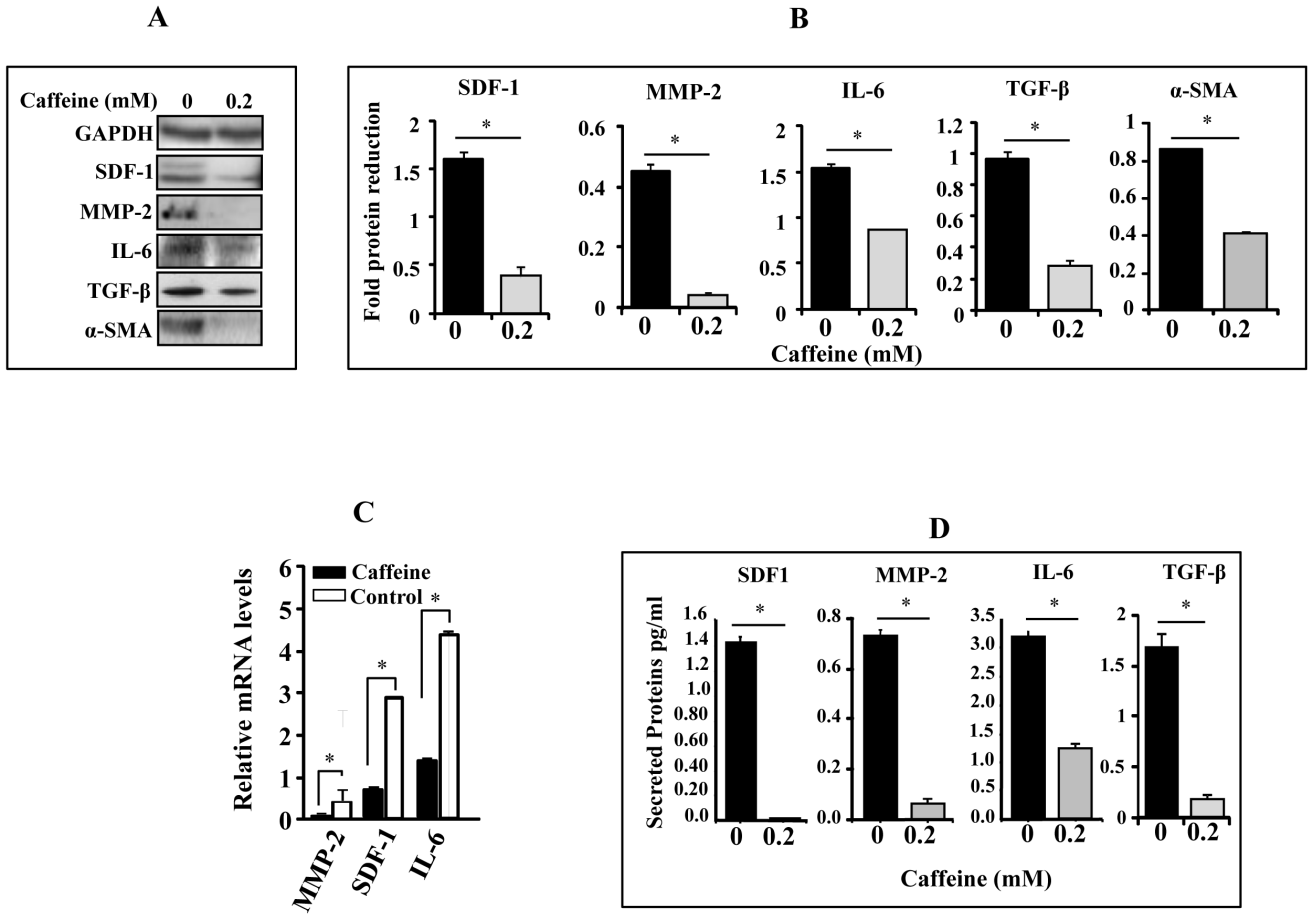
Caffeine inhibits the expression/secretion of SDF1, IL-6, MMP-2 and TGF-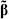. CAF-180 cells were either sham-treated or challenged with caffeine for 1 hr. (A) Whole cell lysates were prepared and used for immunoblotting analysis using the indicated antibodies. (B) Histograms show the expression levels of the indicated proteins. The values were determined by densitometry and normalized against GAPDH. (C) Total RNA was prepared and used to assess the level of the indicated genes by qRT-PCR. The obtained values were normalized against β-actin. (D) Secreted levels of proteins were determined in SFCM by ELISA and shown in the histograms. Error bars represent means ± S.D. *: *p*<0.05.

In order to test the possible caffeine-dependent inactivation of myofibroblasts, we investigated the effect of caffeine on the expression of various markers of active fibroblasts. Therefore, CAF-180 cells were either sham-treated or challenged with caffeine (0.2 mM) for 1 h. Subsequently, cell lysates were prepared and used for immunoblotting analysis using specific antibodies against SDF-1, MMP-2, IL-6, TGF-β and α-SMA. Figure 2A and 2B shows that caffeine significantly suppressed the expression of all these proteins. To confirm this we used quantitative RT-PCR to assess the expression level of these genes at the mRNA level. Figure 2C shows that caffeine treatment (0.2 mM) significantly reduced the mRNA levels of MMP-2, SDF-1 and IL-6.

To further confirm this and show the effect on the secretion of these proteins, serum-free conditioned media (SFCM) was collected from both sham- and caffeine-treated cells (0.2 mM for 1 h), and the levels of secreted proteins were assessed by enzyme-linked immunosorbent assay (ELISA). The levels of secreted proteins from CAF-180 were strongly reduced as compared to the levels of these proteins secreted from the control cells (Fig. 2D). Indeed, the levels of SDF-1, MMP-2, IL-6 and TGF-β were 70, 7.5, 2.6 and 8.75 fold reduced, respectively (Fig. 2D).

### Caffeine Inhibits the Migration/Invasion Potential of CAF Cells through PTEN-Dependent Erk1/2 and Akt Inactivation

**Figure 3:**
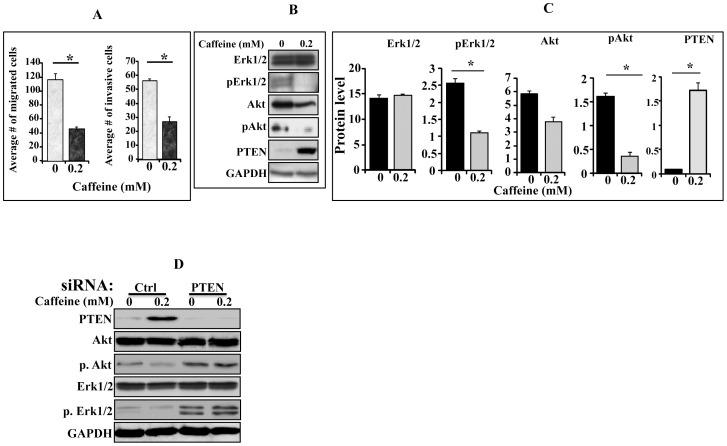
Caffeine suppresses the invasion/migration of active breast stromal fibroblasts through Akt/Erk1/2 inhibition. CAF-180 cells were either sham-treated or challenged with caffeine for 1 hr. (A) Cells were cultured on the upper compartments of BioCoat matrigel chambers in the presence of serum-free media. After 18 hr of incubation, cells were stained with Diff-Quick stain, and then were counted. The histograms depict the average numbers of invasive and migrated cells. (B) Whole-cell lysates were prepared and used for immunoblotting analysis using the indicated antibodies. (C) Histograms show the expression levels of the indicated proteins. The values were determined by densitometry and normalized against GAPDH. Error bars represent means ± S.D. *: *p*<0.05. (D) CAF-180 cells were transfected with PTEN-siRNA or a scrambled sequence, and then cells were either sham-treated of challenged with caffeine for 1 hr. cell lysates were prepared and used for immunoblotting analysis utilizing antibodies against the indicated proteins.

In order to confirm caffeine-dependent inhibition of active fibroblasts, we sought to investigate the effect of caffeine on the migration/invasion abilities of CAF cells. Therefore, CAF-180 cells were cultured in serum-free medium (SFM) and were either sham-treated (control) or challenged with caffeine (0.2 mM) for 1 hr. Subsequently, cells were collected and seeded with SFM into BioCoat Boyden chambers either matrigel-coated (invasion) or uncoated (migration). Complete medium (CpM) was added to the lower chambers of the inserts as chemo-attractant, and cells were reincubated for 18 hr. Figure 3A shows that the migration and invasion of caffeine-treated cells were significantly reduced as compared to control cells (*p* = 0.000178 and 0.000139, respectively).

To explore the molecular mechanisms that underlay caffeine-dependent modulation of stromal fibroblast migration/invasion, we studied the effect of caffeine on the phosphorylation/activation of the pro-invasive protein kinases Erk1/2 and Akt [19], [20]. Therefore, CAF-180 cells were either sham-treated or challenged with caffeine (0.2 mM for 1 hr). Subsequently, cell lysates were prepared and used for immunoblotting analysis using specific antibodies against Akt/pAkt and Erk1/2/pErk1/2. Figure 3B and 3C shows that while the levels of Erk1/2 and Akt were not affected by caffeine treatment, the levels of the active/phosphorylated forms of these proteins were significantly down-regulated in caffeine-treated cells as compared to control cells.

To elucidate how caffeine inhibits the phosphorylation of these 2 protein kinases, we tested the effect of caffeine on PTEN, which is a negative regulator of Akt and Erk1/2 [21], [22]. Figure 3B and C shows that caffeine-treatment markedly increased PTEN protein level (18 fold) as compared to control cells (*P* = 0.0048). These results indicate that caffeine inhibits the migration/invasion abilities of CAF cells through inhibition of Akt/Erk1/2 by PTEN up-regulation. To confirm this, caffeine-related activation of Akt and Erk1/2 was investigated in PTEN-deficient cells. To this end, PTEN was down-regulated using specific siRNA (a scrambled sequence was used as control) in CAF-180 cells, and then both PTEN-deficient and control cells were treated with caffeine (0.2 mM) for 24 hrs. As expected, PTEN was up-regulated in control cells but not in PTEN-siRNA expressing cells (Fig. 3D). However, while the level of active Akt and Erk1/2 decreased in caffeine-treated control cells, their levels remained constant in PTEN-deficient cells (Fig. 3D). This indicates that caffeine-dependent reduction in the level of active Atk and Erk1/2 is PTEN-related.

### Caffeine Suppresses CAF-related Enhancement of Invasion/Migration of Breast Cancer Cells

**Figure 4:**
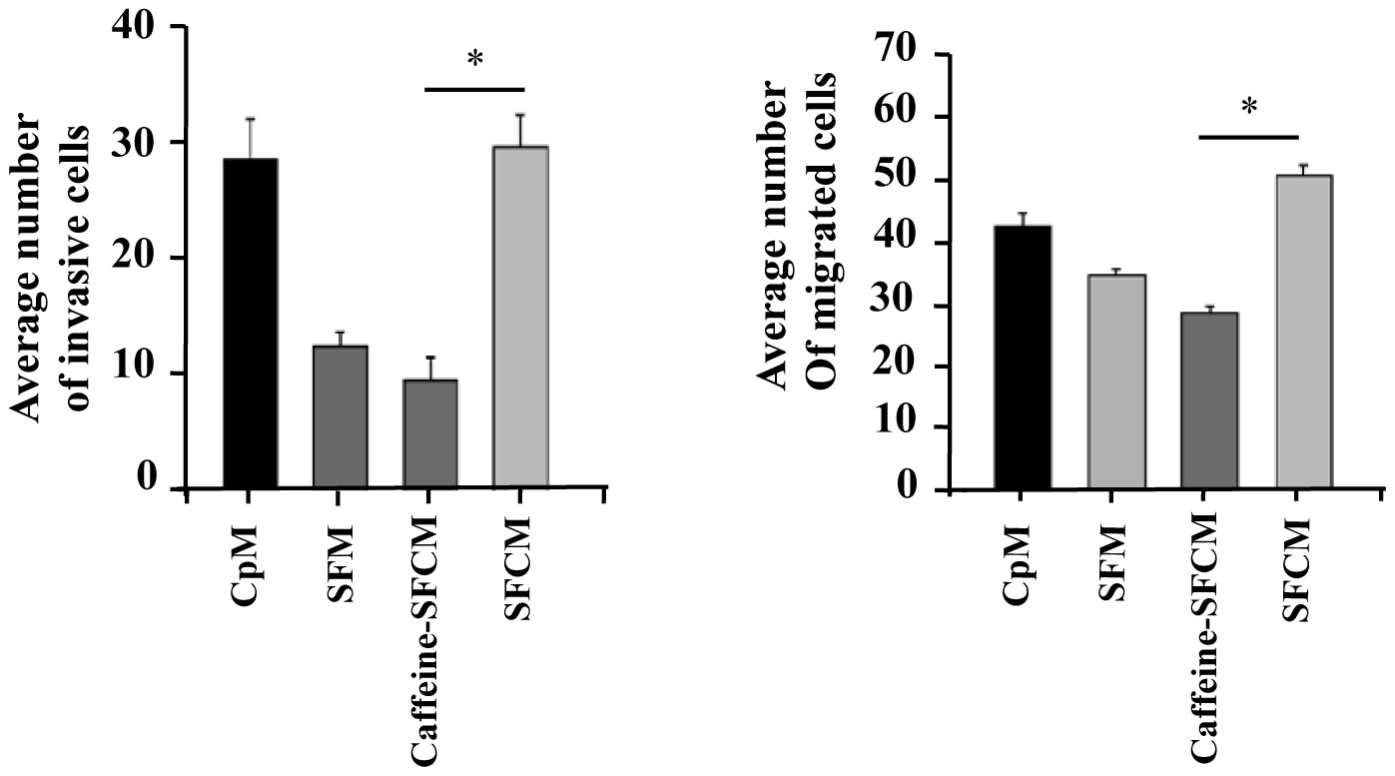
Caffeine inhibits MDA-MB-231 invasion/migration in a paracrine manner. Serum-free conditioned media were collected from CAF-180 cells either sham-treated or challenged with caffeine (0.2 mM, for 1 hr), SFM as well as complete media (CpM) were also used as control. These media were added separately into the lower compartments of the 24-well BD BioCoat plates. MDA-MB-231 cells (1×10^4^) were seeded onto the upper compartment of the migration (non-coated chambers) and invasion (Matrigel Invasion Chamber) plates and incubated for 18 hr. Average numbers of migrated/invaded cells were depicted in the histograms. Error bars represent means ± S.D. *: *p*<0.05.

After showing that caffeine inhibits the secretion of procarcinogenic factors from CAF cells, we sought to investigate whether this inhibition would affect the migration/invasion of breast cancer cells. Therefore, CAF-180 cells were either sham-treated or challenged with caffeine (0.2 mM) for 1 hr, and then caffeine was removed and cells were washed twice with PBS and were subsequently reincubated in SFM for 24 hr to generate SFCM. MDA-MB-231 cells suspended in SFM were added to the upper compartments, while CpM, SFM, SFCM-caffeine and SFCM-control were added separately to the lower compartments of the Boyden chambers used for the migration and invasion assays. After 18 hr of incubation, cells were stained with Diff-Quick stain, and then were counted. Figure 4 shows that the invasion ability of breast cancer cells was 3 fold lower in the presence of SFCM from caffeine-treated cells (caffeine-SFCM) than from control cells (SFCM). Interestingly, the invasion of MDA-MB-231 cells in the presence of caffeine-SFCM was similar to that obtained in the presence of SFM (Figure 4). Similar inhibitory effect was obtained for the migration of MDA-MB-231 cells under the same conditions (Fig. 4). These results show that caffeine suppressed breast stromal fibroblast-dependent induction of the migratory and invasiveness abilities of breast cancer cells, confirming caffeine-dependent inactivation of breast stromal myofibroblasts.

### Caffeine Mediates Sustained Effects

**Figure 5:**
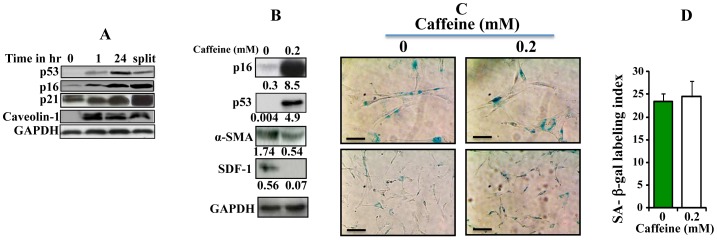
Caffeine effects are persistent. (A) Cells were either sham-treated (0) or challenged with caffeine (0.2 mM, for 1 hr), and then immediately harvested (1) or reincubated in caffeine-free medium for 24 hr, and then were either harvested (24) or split and reincubated for another 48 hr (split). Subsequently, cell lysates were prepared from all these cells and the levels of various proteins were assessed by immunoblotting. (B) Cells were either sham-treated or challenged with caffeine (0.2 mM) for 1 hr, and then reincubated in caffeine-free medium for 24 hr. Cell lysates were prepared and used for immunoblot analysis. The numbers below the bands represent fold of change as compared to the level at time 0, upon normalization against GAPDH used as internal control. (C) SA-β-gal activity was analyzed. Scale bars represent 50 µm. (D) Histogram shows SA-β-gal labeling index. Error bars represent means ± S.D.

After showing that caffeine effect was sustained following caffeine withdrawal and incubation of cells in caffeine-free medium for 24 hr (Fig. 4), we sought to test whether caffeine effect on the various cancer-related molecular pathways is also persistent. To this end, CAF-180 cells were either sham-treated or challenged with caffeine (0.2 mM) for 1 hr, and then caffeine-containing medium was removed and cells were washed 3 times with PBS. Thereafter, cells were incubated in caffeine-free medium for 24 hr, and then were split and reincubated in caffeine-free medium for another 48 hr. Subsequently, cell lysates were prepared and used for immunoblotting analysis using specific antibodies. Figure 5A shows that 1 hr of caffeine treatment increased the levels of the 4 tumor suppressor genes (p16, p21, p53 and caveolin-1). Importantly, this up-regulation was sustained for 24 hr before and 48 hr after splitting in caffeine-free medium. Figure 5B shows that even after 24 hr of caffeine withdrawal the tumor suppressor proteins p16 and p53 were still highly expressed as compared to their basal levels. Concomitantly, α-SMA and SDF-1 protein levels remained also down-regulated (Fig. 5B). This indicates that caffeine treatment for 1 hr led to persistent inhibition of active breast stromal fibroblasts.

One explanation of this phenomenon is the possible caffeine-related induction of senescence (a permanent process) through the induction of p16, p21 and p53. To test this hypothesis, we measured the SA-β-gal activity in caffeine-treated cells (0.2 mM for 1 hr and reincubation for 24 hr in caffeine-free medium). Figure 5C and D shows that caffeine treatment that led to persistent effect, did not change the shape of cells and also did not increase the level of SA-β-gal activity, suggesting that caffeine does not induce senescence in these cells.

### Caffeine Suppresses the Expression/Secretion of VEGF-A in CAFs and Inhibits their Pro-angiogenic Effect

**Figure 6:**
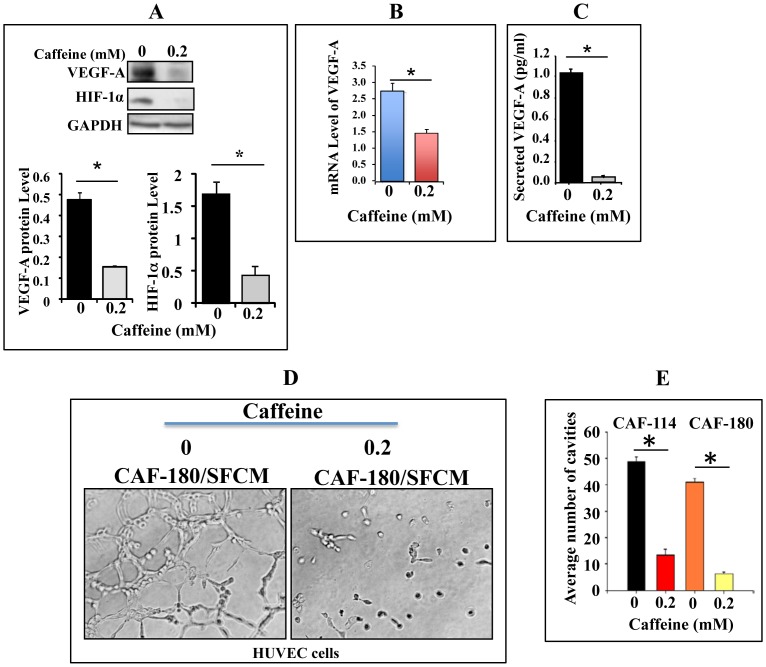
Caffeine inhibits VEGF-A expression/secretion in stromal fibroblasts and their pro-angiogenic effects *in vitro*. CAF-180 cells were either sham treated or challenged with caffeine for 1 hr. (A) Whole-cell lysates were prepared and 50 µg of proteins were used for immunoblotting analysis using the indicated antibodies (upper panel). The histograms show the expression levels of the indicated proteins (lower panels). (B) Total RNA was prepared and used to assess the level of VEGF-A by qRT-PCR. The obtained values were normalized against β-actin. (C) CAF-180 cells were treated with caffeine for 1 hr. Non-treated cells were cultured under the same conditions and were used as control. Secreted levels of proteins were determined by ELISA and shown in the histogram. (D**)** Cells were either sham-treated or challenged with caffeine (0.2 mM) for 1 hr, and then caffeine was removed and cells were washed twice with PBS and were subsequently reincubated in SFM for 24 hr to generate SFCM that have been used to treat HUVEC cells previously plated on matrigel (96-well plate), then incubated at 37°C for 18 hr. (E) Histogram shows the average number of formed cavities. Error bars represent means ± S.D. *: *p*<0.05.

Since CAFs play a major role in the formation of new blood vessels [5], we sought to investigate the possible role of caffeine in the suppression of the main angiogenesis factor VEGF-A in stromal fibroblasts and test their paracrine effect on endothelial cells. To this end, CAF-180 cells were either sham-treated or challenged with caffeine (0.2 mM) for 1 hr, and then cell lysates were prepared and used for immunoblotting analysis using specific antibodies against VEGF-A. Figure 6A shows that caffeine significantly suppressed (3 fold) the expression of VEGF-A as compared to the basal level of the protein (*p* = 0.00555).

To investigate the effect of caffeine on the VEGF-A mRNA, total RNA was purified from the same cells and the *VEGF-A* mRNA was amplified by quantitative RT-PCR (qRT-PCR) using specific primers. Figure 6B shows significant caffeine-dependent down-regulation of the *VEGF-A* mRNA as compared to the control (*p* = 0.01041), indicating that caffeine affects VEGF-A expression at the mRNA level.

Active Akt and Erk1/2 are also major regulators of angiogenesis through the activation of HIF-1α, which up-regulates VEGF-A [23]–[27]. We have shown in Figure 3B that caffeine inhibits Erk1/2 and Akt activation. Therefore, we tested the effect of caffeine on the downstream effector HIF-1α. Interestingly, Figure 6A shows that like for Akt and Erk1/2, caffeine also down-regulated HIF-1-α (3.5 fold). This suggests a potential role of caffeine in inhibiting VEGF-A through suppressing Akt and Erk1/2 and their downstream effector and VEGF-A transactivator HIF-1α.

To further elucidate the inhibitory effect of caffeine on the expression of VEGF-A, SFCM-caffeine and SFCM were respectively collected from caffeine-treated CAF-180 cells and their control cells, and used to assess the secreted level of the VEGF-A protein utilizing the ELISA assay. Interestingly, caffeine markedly suppressed the secretion of VEGF-A from CAF-180 cells. The level of secreted VEGF-A was 21 fold lower from caffeine-treated cells relative to their control cells (Fig. 6C). These results show that caffeine inhibits VEGF-A expression/secretion of active breast stromal fibroblasts.

Next, we investigated the effect of caffeine-treated cells on the differentiation of endothelial cells *in vitro*. Therefore, CAF-180 cells were either sham-treated or challenged with caffeine (0.2 mM) for 1 hr, and then caffeine was removed and cells were washed twice with PBS and were subsequently reincubated in SFM for 24 hr to generate SFCM that have been used to treated HUVEC cells (0.5×10^5^) on Matrigel (96-well plate) for 18 hr at 37°C. Figure 6D and E shows that while the differentiation of HUVEC cells into primitive capillary-like structure occurred in the presence of SFCM, it was strongly inhibited in the presence of caffeine-SFCM. Similar results were obtained using CAF-114 cells (Fig. 6E). Together, these results indicate that caffeine suppresses VEGF-A expression/secretion in active stromal fibroblasts and consequently inhibits their pro-angiogenic paracrine effect.

## Discussion

Cancer is a very complex entity composed of multiple cell types that cooperate to enable tumor growth and spread. Thereby, an efficient treatment should take into account the presence of non-carcinogenic but supportive cells, and hence makes the milieu unfertile for tumor growth. Consequently, agents that can inhibit cancer-stroma crosstalk by normalizing the components of the tumor microenvironment may boost the traditional tumor cell-directed therapy. Therefore, we tested the effect of the nontoxic and pharmacologically safe caffeine in suppressing the carcinogenic effects of active breast stromal fibroblasts. We have shown that caffeine at 200 µM up-regulates the expression of p16, p21, p53 and Cav-1, 4 important tumor suppressor genes with cell non-autonomous tumor suppressor functions [2], [18]. Indeed, these tumor suppressor genes were found to be down-regulated in many CAF cells and were shown to be responsible for the procarcinogenic paracrine effects of these cells [2], [18]. p16 and p53 negatively control the expression/secretion of the major CAF effector SDF-1 [18], [28]. Therefore, caffeine suppressed the expression/secretion of SDF-1 and also MMP-2 and TGF-βfrom CAF cells. Furthermore, caffeine reduced the level of the major marker of myofibroblast cells α-SMA, and strongly inhibited the migratory/invasiveness abilities of CAFs. This effect was mediated through the inhibition of Erk1/2 and Atk. This inhibition was dependent on the up-regulation of their common inhibitor PTEN [29]. Recently, Miwa et al. have shown that caffeine activates PTEN in sarcoma cells leading to the inactivation of Akt. However, caffeine affected the phosphorylation form of the protein with no effect on total PTEN, and 5 mM was required to reach 50% reduction in the level of phospho-PTEN [13]. Similarly, it has been shown that caffeine suppresses NF-κB and Erk1/2 activities in osteosarcoma cells [30]. In another study, 10 mM of caffeine was used to inhibit the Akt pathway in various cancer cells [31]. Together, these results indicate that caffeine can inhibit the Akt pathway. However, we present here the first indication that this inhibition can take place in non-carcinogenic breast stromal fibroblasts and using a concentration as low as 0.2 mM. In another study, Foukas et al. have shown caffeine-dependent inhibition of the kinase activities of PI3Ks *in vitro* [30]. Furthermore, it has been also shown that caffeine can inhibit Akt and Erk in colon cancer cells [32]. This confirms our data showing that caffeine is a strong inhibitor of the motility and the invasiveness of active breast stromal fibroblasts. In addition, we have shown that caffeine reduced the ability of these cells in enhancing the migration/invasion of breast cancer cells *in vitro*, which is a direct consequence of reducing the expression/secretion of different procarcinogenic factors, including SDF-1 and TGF-β. Together, these results indicate that caffeine “normalizes” active breast cancer-associated fibroblasts through up-regulating tumor suppressor proteins and inhibiting the 2 major pro-invasive/−migratory protein kinases Erk1/2 and Akt [19], [20].

Since VEGF-A and its transactivator HIF-1α are major pro-carcinogenic target of p16 and p53 [33], [34], we tested the effect of caffeine on the expression of HIF-1α/VEGF-A and the related pro-angiogenic effect of breast fibroblasts. We have shown that caffeine represses both VEGF-A and HIF-1α in active breast stromal fibroblasts, and consequently repressed their pro-angiogenic paracrine effect (Figure 6). HIF-1α/VEGF-A are also under the control of Akt and Erk1/2, which play major role in angiogenesis [35], [36], and were inactivated by caffeine treatment. This indicates that caffeine suppresses VEGF-A expression and the related angiogenesis through different pathways. Likewise, it has been previously shown that caffeine inhibits adenosine-induced accumulation of HIF-1α and VEGF-A in colon cancer cells [32]. Furthermore, chick chorioallantoic membrane assays were used to show that caffeine inhibits angiogenesis and the proliferation of HUVEC cells [37]. In addition, Li et al have recently shown caffeine-dependent induction of endothelial cell death and the inhibition of angiogenesis [37]. Notably, in these 3 studies and others the effect of caffeine was direct on endothelial cells, while in the present report the effect of caffeine was indirect through the inhibition of breast stromal fibroblasts pro-angiogenic paracrine effect. This shows that caffeine possesses anti-angiogenic effect through both autocrine and paracrine manners. This suggests that caffeine could be of great value for cancer prevention and/or treatment by preventing the pro-vascularization effect of stromal myofibroblasts.

Importantly, we have also shown that all the effects of caffeine on CAF cells were sustained even after caffeine removal and cell splitting. This provides a proof of principle that cancer-associated breast stromal fibroblasts can be inactivated, and that caffeine can trigger this through modulating several cancer/inflammation-related pathways. The permanent nature of caffeine effect could result from epigenetic alterations. This effect could be mediated through modulating the expression of genes capable of affecting DNA methylation, such as DNMT, p16 and IL-6. Indeed, it has been previously shown a causal role of p16 disruption in modulating DNA hypermethylation, and the role of IL-6 in controlling methylation in breast cancer cells [38], [39].

While the role of caffeine in breast cancer prevention is still controversial, several reports have shown clear inverse association between caffeine-containing beverages and risk of postmenopausal breast cancer [40]. Interestingly, it has been shown in a recent study on 93,676 Caucasian women that consumption of caffeinated coffee was associated with reduction in non-melanoma skin cancer [41]. Furthermore, caffeine inhibited the development of benign mammary gland tumors in 7,12-dimethylbenz(a) anthracene (DMBA)–treated female Sprague-Dawley rats [42]. Caffeine has also been reported to suppress metastasis in a transgenic mouse model of mammary tumors [43]. Moreover, in SKH-1 mice at high risk of developing malignant and nonmalignant tumors, oral administration of caffeine for 18–23 weeks inhibited the formation and decreased the size of both nonmalignant and malignant tumors [44]. Therefore, caffeine or another non-toxic inhibitor of the STAT3 pathway could constitute an efficient approach to normalize active breast stromal fibroblasts and hence participates in efficient eradication of tumor cells through targeting their supportive milieu.

A cup of coffee contains approximately 100 mg of caffeine [45], indicating that micromolar concentrations of caffeine can be reached in the human circulation by the daily coffee consumption. Indeed, it has been shown that oral absorption of caffeine (5 mg/Kg) was very rapid, reaching a peak plasma concentration (15.9–18.7 µg/mL) after approximately 30 min. Interestingly, the caffeine plasma half-lives varied from 2.7 to 9.9 h, indicating substantial inter-subject variability in caffeine elimination [46]. This indicates the possibility of reaching physiologically active amount of caffeine in human circulation with the consumption of reasonable amount of caffeine.

In summary, our study shows that caffeine can normalize active breast stromal fibroblasts, and therefore suppresses their procarcinogenic/metastatic potential. This effect is mediated through the induction of important tumor suppressor proteins (p16, p21, p53 and Cav-1), which suppresses the secretion of various procarcinogenic cytokines.
